# Cyanidin-3-*O*-glucoside: Physical-Chemistry, Foodomics and Health Effects

**DOI:** 10.3390/molecules21091264

**Published:** 2016-09-21

**Authors:** Francisco J. Olivas-Aguirre, Joaquín Rodrigo-García, Nina del R. Martínez-Ruiz, Arely I. Cárdenas-Robles, Sandra O. Mendoza-Díaz, Emilio Álvarez-Parrilla, Gustavo A. González-Aguilar, Laura A. de la Rosa, Arnulfo Ramos-Jiménez, Abraham Wall-Medrano

**Affiliations:** 1 Instituto de Ciencias Biomédicas, Departamento de Ciencias Químico-Biológicas, Universidad Autónoma de Ciudad Juárez, Anillo Envolvente del PRONAF y Estocolmo s/n, Ciudad Juárez 32310, Chihuahua, Mexico; Javier_Olivas22_09@hotmail.com (F.J.O.-A.); Jogarcia@uacj.mx (J.R.-G.); nmartine@uacj.mx (N.d.R.M.-R.); ealvarez@uacj.mx (E.Á.-P.); ldelaros@uacj.mx (L.A.d.l.R.); aramos@uacj.mx (A.R.-J.); 2 Departamento de Investigación y Posgrado en Alimentos, Facultad de Química, Universidad Autónoma de Querétaro, Cerro de las Campanas s/n, Querétaro 76010, Querétaro, Mexico; smendoza@uaq.mx (A.I.C.-R.); cardenas.arely.i@gmail.com (S.O.M.-D.); 3 Coordinación de Tecnología de Alimentos de Origen Vegetal, Centro de Investigación en Alimentación y Desarrollo, AC. Carretera a la Victoria km. 0.6, AP 1735, Hermosillo 83000, Sonora, Mexico; gustavo@ciad.mx

**Keywords:** anthocyanin, cyanidin 3-*O*-glucoside, cyanidin, antioxidant, bioaccessibility, berries, phenolic compounds, foodomics, splanchnic metabolism

## Abstract

Anthocyanins (ACNs) are plant secondary metabolites from the flavonoid family. Red to blue fruits are major dietary sources of ACNs (up to 1 g/100 g FW), being cyanidin-3-*O*-glucoside (Cy3G) one of the most widely distributed. Cy3G confers a red hue to fruits, but its content in raspberries and strawberries is low. It has a good radical scavenging capacity (RSC) against superoxide but not hydroxyl radicals, and its oxidative potential is pH-dependent (58 mV/pH unit). After intake, Cy3G can be metabolized (phases I, II) by oral epithelial cells, absorbed by the gastric epithelium (1%–10%) and it is gut-transformed (phase II & microbial metabolism), reaching the bloodstream (<1%) and urine (about 0.02%) in low amounts. In humans and Caco-2 cells, Cy3G’s major metabolites are protocatechuic acid and phloroglucinaldehyde which are also subjected to entero-hepatic recycling, although caffeic acid and peonidin-3-glucoside seem to be strictly produced in the large bowel and renal tissues. Solid evidence supports Cy3G’s bioactivity as DNA-RSC, gastro protective, anti-inflammatory, anti-thrombotic chemo-preventive and as an epigenetic factor, exerting protection against *Helicobacter pylori* infection, age-related diseases, type 2 diabetes, cardiovascular disease, metabolic syndrome and oral cancer. Most relevant mechanisms include RSC, epigenetic action, competitive protein-binding and enzyme inhibition. These and other novel aspects on Cy3G’s physical-chemistry, foodomics, and health effects are discussed.

## 1. Introduction

*“An apple a day keeps the doctor away”* is a premise that alludes to the fact that many non-communicable chronic diseases (NCCD) could be prevented with a sufficient daily intake of bioactive molecules from fruits and vegetables. Soluble and insoluble dietary fiber, antioxidants, functional carbohydrates and polyunsaturated fatty acids, among others, individually or in a concerted action are responsible for many beneficial health effects. Particularly, dietary antioxidants (AOX) such as pro-vitamins and phenolic compounds (PC), including anthocyanins (ACNs; *anthos* = flower, *kianos* = blue), consumed daily will alleviate the oxidative stress associated with many molecular events within our bodies [1,2]. A strong body of evidence supports other ACNs’ bioactivities including anti-inflammatory, neuro-protective, anti-microbial, anti-viral, anti-thrombotic and epigenetic actions [3,4,5,6].

However, not all ACNs are equal [7], neither is their specific metabolic fate and bioactivity. Many physical and chemical factors present in natural or prepared plant foods [8,9,10,11] along with several physiological barriers within our body [12,13,14], could restrain their metabolic action. In fact, the nutraceutical potential of ACNs is structure-specific, an aspect associated with their specific physicochemical behavior within foods and biological systems [15]. In this sense, important pieces of the Cyanidin-3-*O*-glucoside (Cy3G; *A.K.A.* chrysanthemin, kuromanin) puzzle, the most widely distributed anthocyanin in edible fruits [16,17], have been published in the last 10 years and some of which are discussed in the following paragraphs.

## 2. Physical Chemistry of Cy3G

A correct understanding of Cy3G’s bioactivity requires knowing its structural features and physicochemical behavior. For example, its binding potential and radical scavenging capacity (RSC) depend on its REDOX behavior while its absorption and metabolic fate within the gastrointestinal (GI) tract relies on the presence of glucose and/or other glycoside moieties. Pure Cy3G is susceptible to degradation by many physicochemical factors including pH, light, oxygen, solvents, temperature and metal ions [11,18,19]. From a technological perspective, this justifies why ACNs-based products are not widely used as pigments [20] since they are unstable during storage [21]. Also, Cy3G is exposed to many factors along the GI tract (pH, ionic strength), which affect its bioaccessibility, bioavailability and further bioactivity [22,23]. In the following section, some of these features are reviewed.

### 2.1. Chemical Structure

**Figure 1:**
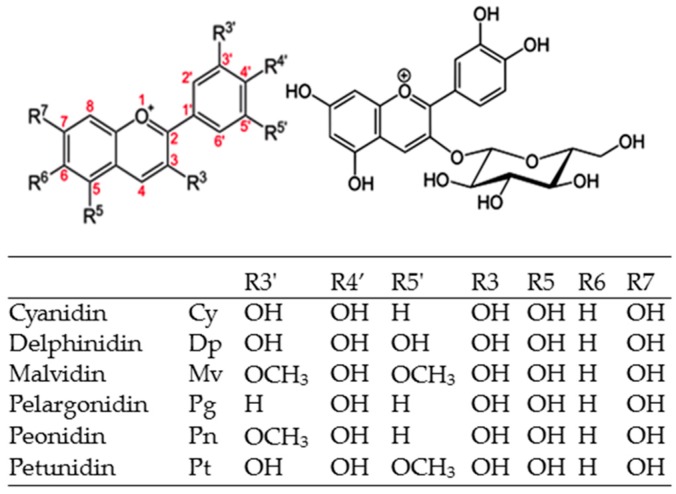
Structure of common anthocyanidins (**left**) and Cyanidin-3-*O*-glucoside (Cy3G; **right**).

ACNs are anthocyanidin glycosides. Their backbone consists of a benzopyran core [benzoyl ring (A), pyran ring (C)], a phenolic ring (ring B) attached to its (2-position and a sugar moiety mainly at its 3-position in the C-ring (Figure 1). 31 anthocyanidin (aglycones) and more than 600 ACNs have been identified to date [16]. However, 90% of all naturally occurring ACNs are based on six aglycones differing in their B-ring substitution pattern: cyanidin (Cy) about 50%, delphinidin (Dp), pelargodin (Pg) and peonidin (Pn) about 12% each and petunidin (Pt), malvidin (Mv) about 7% each. These aglycons are further classified by the nature and number of bonded sugars and the presence of aliphatic or aromatic carboxylates (attached to their sugar moieties) [10,24], with 3-monosides (mainly glucosides), 3-biosides, 3,5- and 3,7-diglucosides from the 3 non-methylated aglycones (Cy, Dp and Pg) the most common.

From an analytical standpoint, the structural diversity of ACNs represents a challenge for their isolation and identification [10,21]. However, the mass spectral fingerprint of Cy and many of its naturally occurring glycosides have been established so far [14,16,17,25], some included in Table 1.

The pattern of glycosylation and methylation influences Cy’s hydrophobic (octanol)/hydrophilic (water) partition coefficient (LogP), its polar surface area (Å^2^) and its molecular weight (MW), all having important implications in the metabolic fate of Cy-derivates (ADME: Absorption, Distribution, Metabolism and Excretion) [26]: Cy has a lower MW (287.24 g/mol) and Å^2^ (114.3) and is less hydrophilic (LogP = 3.05) than Cy3G (449.4 g/mol, Å^2^ = 191, LogP = 0.39; Table 1). A second glycosylation (Cy-3,5*-O-*diglucoside, Cy3,5GG) increases its hydrophilic nature but that compromises its absorption capacity [27] while an extra malonyl group (Cy3MG) does the opposite [28]. Other structural features in Cy3G also have important implications on its chemical reactivity in vitro. Fernandes et al. [9] used STD-NMR spectroscopy and molecular dynamics simulations to show that the absence of an extra hydroxyl at R^5’^ in Cy3G (Figure 1) affects its binding capacity toward citrus pectins when compared to Dp3G. Also, Phan et al. [29] demonstrated that Cy3G (as flavylium cation, pH 3.4) binds spontaneously within 1 min to bacterial (*Gluconacetobacter xylinus* ATCC 53524)-derived cellulose, steadily increasing up to 2 h. They reported that this binding behavior is not limited by the available interacting sites in cellulose but to the amount of free Cy3G molecules, proposing a Langmuir binding isotherm model (Equation (1)):

Q = Q_max_ × [(*K*_L_·C) × (1 + *K*_L_ × C)^−1^] (1)

where Q is the amount of absorbed Cy3G per unit mass of cellulose (µg·mg^−1^), Q_max_ is the apparent maximum adsorption capacity (1109 µg·mg^−1^ of cellulose), *K*_L_ is the apparent binding affinity constant and C is the free Cy3G concentration at equilibrium (mM).

By applying this equation, a “Cy3G saturation effect” can be observed at about 200 mM. Also, Oliveira and Pintado [30] using an in vitro model to simulate GI conditions demonstrated a “bind-release” behavior between Cy3G and pectin/chitosan at each digestion step (oral, gastric, and intestinal), suggesting a protective mechanism of this polymeric mixture over Gy3G degradation since Cy3G is progressively released from protein and polysaccharide bonds, which are available for its potential absorption by GI epithelial cells.

It is noteworthy that Cy3G also binds to proteins in vitro. In the same experiment reported by Oliveira and Pintado [30], an even stronger binding capacity of Cy3G toward P/C+β-lactoglobulin was observed. Tang et al. [31] reported the differential binding capacity of three ACNs to human serum albumin (HSA; Dp3G> Cy3G> Pg3G) but their capability to induce structural changes in this protein was different (Pg3G> Cy3G> Dp3G). Tang et al. [32], by using multi-spectral techniques and molecular modeling, suggested that Cy3G–protein interactions are established by hydrogen bonding and van der Waals forces and, as a consequence, the secondary structure of bovine serum albumin (BSA), hemoglobin (Hb), and myoglobin (Mb) is partially destroyed (less% α-helixes). These molecular interactions have important implications in Cy3G transport in the bloodstream (HSA) but could also affect the correct functionality of heme-containing proteins (Hb, Mb). Cy3G is commonly represented as a cation (Figure 1), which is only possible under acidic conditions such as in gastric juice, and in silico assays have revealed that cationic Cy3G cannot be absorbed through passive diffusion [33]. However, a simple substitution at R3′ [–H (Pg3G) by –OH (Cy3G)] modifies Cy3G bioaccessibility, absorptivity, and metabolism within enterocytes [34].

Lastly, rare anthocyanidins such as 3-deoxy-anthocyanidins, hydroxylated at the 6th position, 5, 7, 3′, 5′-*O-*glycosilated, *C*-glycosylated, or aliphatic (mainly malonic and pyruvic acids)- or PC-acylated ACNs, which are also currently studied [9,10,28,35] because they seem to be more bioactive than conventional counterparts. For instance, Cy-malonyl-glucoside (Cy-Mal-3G) possesses a stronger anti-cancer (colon, liver, prostate, and breast) activity than Cy3G [36]. Also, Cy3G acylated with lauric acid improves its stability because an ester group is more stable than a hydroxyl group [37]. However, the formation of Cy3G adducts with pyruvic acid during wine ageing or fruit juice processing reduces (about 10 times) its radical RSC toward the superoxide anion [38].

### 2.2. Color

The color of ACNs-rich fruits is a matter of quantity (biosynthesis), molecular inter-play and physicochemical stability. In particular, production and stability of red Cy3G in plants has been extensively studied in horticultural sciences, an aspect that will be further discussed in this article. Many structural features, such as the number of hydroxyl groups, their degree of methylation and the nature and number of sugar moieties bound to the molecule, are related to the color of ACNs. In nature, ACNs show great color diversity from yellow (480 nm) to red (730 nm), and, particularly, Cy3G confers a red hue to fruits [27]. However, the maximum absorption [λ_max_ (εmol) nm] of its flavylium (2-phenyl-1-benzopyrilium) nucleus [10] is more restricted to the six most common aglycones and is related to their B-ring hydroxylation pattern: Cy and Pn (516 nm), Pg (520 nm), and Dp, Pt, Mv (546 nm).

The color stability of ACNs depends on their structure, pH, temperature, light and the presence of complexing agents such as PC and metals [21]. The simple attack by water or sulfites converts the flavylium ion into a colorless pseudobase (nucleophilic addition). However, the color of ACNs also depends on their interactions with other molecules via hydrogen bonds or via “hydrophobic vertical stacking” which is a combination of van der Waals and hydrophobic forces between the planar flavylium and another planar molecule to form a π-π complex. Color enhancement [hyperchromic effect (Δ*A*) + bathochromic shift (Δλ)] and a stabilization phenomenon called co-pigmentation, results from several inter-molecular associations: (i) between two identical ACNs (self-association); (ii) between one of its aromatic substituents (intra-molecular co-pigmentation) with another non-colored molecule (intermolecular co-pigmentation) or; (iii) with a metal ion, forming π-π and other complexes in solution [10]. For example, Bakowska et al. [39] reported a Δλ = 21.4 nm and Δ*A* = 0.48 with complexes of Cy3G and flavones isolated from *Scutellaria baicalensis Georgi*, a Chinese herb used to treat bacterial infections of the respiratory system and GI tract. Pacheco-Palencia [21], when evaluating the influence of the external addition (ratio 1:10 *w*/*w*) of rooibos tea (rich in flavone-C-glycosides) to commercial rosemary extracts on the stability of two açai-derived ACNs [40% Cy3G (40%) + 60% Cy-3*-O-*rutinoside (Cy3R)] model solutions (500 mg/L), found an Δ*A* = +18% with no changes in Δλ. However, co-pigmentation depends on the ortho-dihydroxyl arrangement in the B-ring in such a way that Cy, Dp, and Pt have the ability to form such complexes, but Mv, Pg or Pn do not [40].

### 2.3. Temperature

Several industrial processes, such as dyeing of fabrics and food product manufacturing, apply high temperatures to raw/purified sources of ACNs [41,42]. For example, Manosur et al. [20] evaluated the effect of dye bath pH and temperature on the color of wool fabrics by an aqueous extract of *Vitis vinifera* L. leaves rich in ACNs [acetylated ACNs (69.2%) > Dp3G (10.1%) > Pn3G > Mv3G > Cy3G (3.27%)] showing that processing at 45 °C and 95 °C results in red and brown shades of dyed fabrics, respectively. Also, conventional or microwave (300W) heating at 100 (atmospheric), 38.5, and 7.3 kPa used to concentrate pomegranate juice reduces its Cy3G content but its di-glucoside (CyGG) is not much affected. Also, Mildner-Szkudlarz et al. [43] evaluated the effect of replacing raspberry pomace (0%, 10% and 20%) into wheat flour muffins prepared under various baking conditions (140 °C/30 min, 180 °C/20 min, 240 °C/15 min) showing that low temperatures but longer baking time decrease the amount of Cy3G, independently of the initial percentage incorporated. Slavin et al. [44] found same results when baking black and yellow soybean crackers.

A recent review on the mechanisms of degradation during high thermal processing of ACNs-rich foods [11] indicates that several heating operations (e.g., blanching, pasteurization) and their duration can markedly affect the ACNs content in fruits and vegetables, resulting in a variety of chemical species depending upon the severity and nature of heating. Particularly, Cy3G degradation involves de-glycosylation and further cleavage of covalent bonds leading to phloroglucynaldehyde (PGA) and 4-hydroxybenzoic acid under isothermal conditions. However, ACNs’ degradation follows a non-isothermal kinetic behavior in liquid (concentrated juices) solid (fruits) and semisolid (pomances) food systems. Fortunately, ohmic-, dielectric-, radio frequency- or, microwave-heating, are promising non-thermal technologies that may reduce ACNs loses from prepared foods [45]. The temperature of storage also affects Cy3G stability. Pacheco-Palencia and Talcott [21] evaluated this effect at 5, 20 and 35 °C in Cy3G (40%) /Cy3R (60%) based solutions (500 mg/L), showing that temperature significantly affects Cy3G stability (Equation (2)) and despite there is an important hyperchromic effect (Δ*A*) due to the addition of phenolic acids, flavone-C-glycosides or procyanidins, they do not protect Cy3G degradation from 20 to 35 °C.

Half-Life (days) = 12.6e^−0.1034(T°C)^ (2)

### 2.4. pH

At room temperature and minimally acidic conditions, Cy3G exist in four species in equilibrium [22,46]. At more acidic conditions (pH ≤ 4.0), Cy3G exist primarily in the form of flavylium cation (red); as pH increase increases it is transformed to either its carbinol (hemiketal) form (colorless, pH 5.2) as a result of hydration of the flavylium cation or to its quinoidal form (blue color, pH 5.5–6.0) by means of a proton loss. Finally, these species reach an equilibrium through tautomerization forming an open *cis*-chalcone (light yellow, pH >6.0). In vitro, each form has a different reactivity and antioxidant capacity, since more hydroxyl groups (e.g., hemiketal > quinoidal) means more antioxidant activity [47].

Sui et al. [8] evaluated the stability of Cy3G and Cy3R from black rice in an aqueous system within a pH range from 2.2 to 6.0 and a temperature range from 100 °C to 165 °C. Cy3G was more susceptible to pH or temperature than Cy3R but pH played an important role in stabilizing both molecules under thermal treatment. However, Pacheco-Palencia and Talcott [21] did not find such stabilizing effect at storing temperatures (5, 20 and 35 °C) and nearby its flavylium cation form (pH = 3.0, 3.5, 4.0). Nevertheless, processing wool fabrics with extracts from *Vitis Vinifera* L. leaves at a high temperature but under acidic conditions improves their color strength [20]. The most profound effect of pH is surely the loss of its RSC, which is discussed below.

### 2.5. Antioxidant Capacity

An important biological effect of ACNs is their antioxidant capacity, which could be affected by many of the aforementioned factors. This property has been related to the prevention of inflammatory conditions, cardiovascular disease (CVD) and cancer [10]. The antioxidant protection mechanism includes: A) quenching singlet oxygen (^1^O_2_), B) scavenging reactive oxygen species (ROS), C) chelation of trace metals involved in free radical production or, D) inhibition of ROS-promoting enzymes [48]. When compared to other flavonoids, ACNs are very efficient quenchers of singlet oxygen: one molecule of ACNs is degraded (by its flavylium cation) by every 125 molecules of ^1^O_2_ quenched; the biological relevance relies on the fact that ACNs can be available for many consecutive processes [49]. However, the structural modifications (e.g., type of glycosylation) of ACNs play an important role on the observed antioxidant activity. For instance, the oxidation potential for Cy3G (500 mV vs. Ag/AgCl) is shifted towards a more positive value when compared to its aglycone (Cy) alone (403 mV vs. Ag/AgCl). The more positive oxidation value the less the antioxidant power [50]. The effect on the shift of the oxidation potential has been ascribed to the “steric hindrance” of the glucose moiety in C-ring, which reduces B-ring coplanarity with the rest of the molecule, decreasing the conjugation [51,52].

In general, ACNs have a strong RSC and their electrochemical oxidation shows more than one oxidation process. Three oxidation processes have been reported for Cy3G (Table 2): Electron oxidations in the catechol group (B-ring), the resorcinol group (A-ring) and during the opening of C-ring of Cy3G hemiketal form [47,53]. When a deglucosylation occurs in Cy3GG an oxidation peak potential corresponding to A-ring oxidation occurs at higher values [47].

It is noteworthy that Cy3G oxidative potential is pH-dependent in such way that as pH increases potentials shift to lower values (approximately 58 mV per pH unit [53]. However, Cy3G is easily oxidized at higher pH values due de-protonation of the hydroxyl groups [47,52]. Also, Garcia-Alonso et al. [38] reported that the RSC toward O_2_^−^ of several ACNs was Mv3G (about 13.3 μM) >Dp3G >Pt3G >Pg3G >Cy3G (33.3 μM) but for ^•^OH was Pg3G>Dp3G >Pt3G >Cy3G >Mv3G at a higher concentration (400–1000 μM); this indicates that the amount of OH^−^ and nature of substitution in B-ring, are important determinants of the RSC of ACNs. As an example, the absence of catechol group seems to affect its activity [54] while the presence of hydroxyl groups at the 3 and 5 position seems to increase its activity [55]. From a physiological stand point, it is generally accepted that flavonoids (and particularly ACNs) have low plasmatic antioxidant activity, this because of that as per aforementioned and its fast metabolism. The maximum plasmatic antioxidant value seems to be reached quickly (between 15 and 30 min) [56] before consumption with important losses (almost all) of its activity in the first plasmatic hour. Pro-oxidant activities have been reported for C3G [54] between 0 and 400 µM (nonexclusive event for ACNs), however, it still remains unclear. However, this event seems to be related to the bioaccessibility, bioavailability and absorption capacity of ACNs within/from the GI milieu, their stability in food products and their bioactivity [57].

## 3. Cy3G in Plant Biochemistry

Once the many structural and physicochemical limitations of Cy3G in vitro are known, the factors associated to its sufficient intake (convenient plant sources) and in vivo metabolism (consumer’s GI tract) should be studied. Together, these factors are responsible for the ultimate fate and bioactivity of Cy3G in target tissues. First, it is essential to examine its biosynthesis in edible plants which in turn justify their richness in Cy3G. Second, the conflicting results from a plethora of epidemiological and case-control studies involving Cy3G-rich foods or supplements and its health effects could be explained in the context of Cy3G splanchnic metabolism.

### 3.1. Biosynthesis

ACNs are produced and accumulated in different plant organs in response to several environmental (e.g., stress), genetic (e.g., senescence) and developmental (e.g., ripening) factors [10,37,58]. For example, biosynthesis of ACNs protects certain rice cultivars from drought stress by exerting many antioxidant mechanisms [59]. ACNs are also related to other activities within plants such as visual signals and as antimicrobial agents [57,60]. A comprehensive review on the multiple functional roles of ACNs in plant–environment interactions has been recently published by Landi et al. [61].

**Figure 2:**
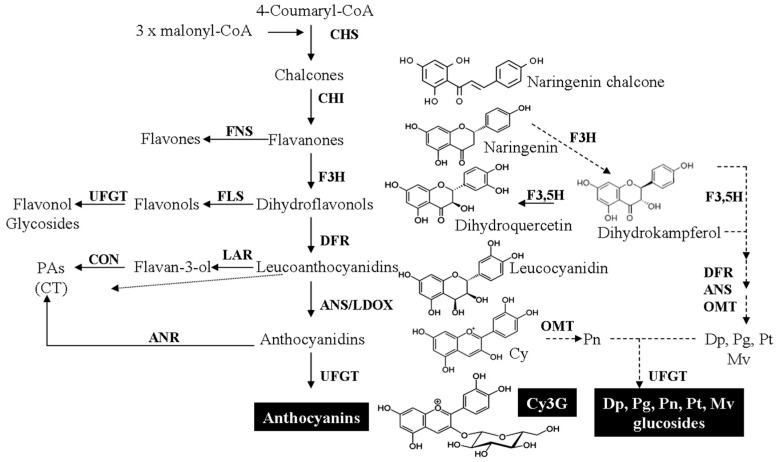
Anthocyanin biosynthesis. Cy3G main steps and crossroads. See abbreviations section for the meaning of each term.

Cy3G is produced through the flavan-3-ol pathway (Figure 2).

Initially, one molecule of 4 (p) -coumaryl-CoA (coming from phenylalanine) is condensed with three molecules of malonyl-CoA (coming from Acetyl-CoA) to get one naringenin chalcone (tetrahydroxychalcone), a reaction catalyzed by chalcone synthase (CHS). The accumulation of this intermediary in plant tissues is quite rare and so it is further transformed into 2*S*-naringenin (flavanone) by chalcone isomerase (CHI). In the 1st crossroad in ACNs synthesis, 2*S*-naringenin can be either directed to flavone synthesis [by flavone synthase (FNS)] or hydroxylated by flavanone-3-hidroxylase (F3H) to produce dihydrokampferol (dihydroflavonol) and this molecule can be either directed to flavonol synthesis (2nd crossroad) by flavonol synthase (FLS) or ACNs’ synthesis by transforming it to a leucocyanidin Dihydroflavonol-4-reductase (DFR) and further by leucoanthocyanidin reductase (LAR) for falvan-3-ol/proanthocyanidin synthesis (3rd cross road).

Dihydrokampferol can be converted into three different intermediary molecules leading to all six common anthocyanidins [62]: (A) to dihydroquercetin catalyzed by flavanone 3′-hydroxylase (F3′H) which further gives Cy and Pn; (B) to dihydromyricetin catalyzed by flavanone 3′,5′-hydroxylase (F3′,5′H) which further gives Dp, Pt and Mv and; (C) to leucopelargodin no additional hydroxylations on the B ring will eventually lead to Pg. These events involve the following enzymes upon request: Anthocyanidin synthase (ANS) also known as leucoanthocyanidin dioxygenase (LDOX), O-methyltransferase (OMT). Finally, all proanthocyanidins (aglycones) are glycosylated by UDP-glucose-flavonoid-3*-O-*glucosyltransferase (UFGT). It is noteworthy that, ANS catalyzed reaction confers a formal positive charge to the pyran ring, while UFGT is specific to the substitution position [10]. Cy3G can further undergo other structural transformations (e.g., acylation/glycosylation) increasing the diversity of Cy3G derivatives [7,63].

ACNs’ profiles and consequently Cy3G’s natural occurrence depend on the expression of genes involved in their biosynthetic pathway and are characteristic of a particular plant family/species and a specific plant part [10]. According to Zhao et al. [37], two set of genes are needed for ACNs biosynthesis: Structural genes (pathway enzymes) and those encoding their transcription factors (regulatory proteins: MYB, basic Helix-Loop-Helix and WD40). Variations in color intensity can be attributed to different expression patterns. For example, the tight control of MYB over *UFGT* gene is responsible for the absence of ACNs in white grapes [64], Malay apples [65] and yellow pears [66], and pelargodin-ANS (PgANS) seems to be the limiting enzyme in white pomegranate [37] while mutations in a gene encoding anthocyanidin-3-glycoside rhamnosyl-transferase (3RT) results in black berry cultivars with a lower than normal ACNs content [63]. The concerted action of regulatory and pathway genes also responds to stressors and the ripening process. Kovinich et al. [7] reported that *Arabidopsis thaliana* preferentially synthesize different Cy3G-derivates characterized by multiple glycosylations/PC acylations in response to distinct stresses during growing (High MgSO_4_, no phosphate, pH 3.3 or high sucrose), suggesting that each Cy3G derivate imparts a function favorable in a particular stress condition. On the other hand, Song et al. [66] reported that *ANS, CHI, F3H, DFR, UFGT* and other proteins such as cytochrome c and cytochrome c oxidase subunit 2 all significantly increases during ripening of strawberry fruits (“Honeoye” and “Mira”).

Lastly, novel breeding and biotechnological strategies applied to ACNs-rich fruit cultivars may enhance their nutritional value and nutraceutical potential [67]. Two research lines are currently carried on: (A) new cultivars with improved levels of functional phytochemicals and (e.g., high-throughput technologies for plant genotyping to select improved berry cultivars); and (B) nutrigenomic actions in humans and animal models (e.g., the effect of eating ACNs-overexpressing berries on the expression of cytoprotective genes such as Nrf2. The health effects of these richest ACNs sources will constitute the next generation of scientific studies.

### 3.2. Dietary Sources

Due to the limited occurrence of anthocyanidins (aglycons) and glycosylation/acylation patterns in edible plants, it is not surprising a more restricted ACNs distribution within fruits and vegetables for human consumption. In this sense, Cy3G is one of the most common [68] but rarely the major CAN; exceptions to this rule are black elderberry, blue hybrid maize and Korean black raspberry. The daily intake and further bioactivity of Cy3G depends largely on the proper selection of their plant sources, as concluded from the previous section. For instance, a higher consumption of conventional (e.g., pomegranate, blackberry) or exotic (e.g., bilberry, elderberry, mulberry) red-to-blue fruits should be recommended. However, ACNs’ (particularly Cy3G) food composition tables are still rather limited [69]. Table 3 summarizes the richness in Cy3G of some edible sources reported in the *Phenol Explorer 3.6* database and from other sources of information [36,43,70] discussed in this article. Despite the fact that Cy3G is widely distributed in fruits (mainly in berries and other blue and red fruits and vegetables), it is not necessarily the main ACNs. For instance, strawberry has 15 times more Pg3G and raspberry (fresh/pomace) 1.4–1.5 times more Cy-3*-O-*sophoriside (Cy3So) than Cy3G.

The specific Cy3G profile in any plant food could be affected by several factors including the varietal and ripening stage. For example, Niño-Medina et al. [71] evaluated the ACNs profile of different eggplant (*Solanum melongena* L.) cultivars grown in a same location, showing that a Philippine varietal had the highest ACNs content (161.10 mg Cy3G equivalents/100 g FW) and the higher RSC (92.5% inhibition of DPPH radical) as compared to other Chinese, American, Hindu and Thai varieties. Song et al. [66] reported important changes in Cy3G (0.008/0.015–0.69/0.48 mg/100 g FW) and Pg3G (0.23/0.15–47.07/31.39 mg/100 g FW) content in strawberry fruit (‘Honeoye’ and ‘Mira’) at different ripening stages (white-to-red). Also, major ACNs in red pomegranate (skin and aryls) are Cy3G, Pg3G and Cy3GG, all of them affected by the ripening process but white pomegranate do not have detectable levels of any ACN [37].

Dietary surveys with detailed information on total and specific intake of ACNs are also scarce. From the very few national surveys, a conclusion can be drawn: the daily intake of ACNs and particularly Cy3G do not depend on the richness of their sources. *Per capita* daily intake (mean) of ACNs was estimated to be 12.5 mg/day in US adults in 2000–2002 [16], 80% coming from blueberry, grape, onion, grape 100% juices, raspberry, red cabbage, wine and cherry sweet [17,72]; in 2007–2008, this intake was 11.2 mg/day adding banana, red/purple vegetables and yogurt to the list [73]. In Europeans, mean intake of total anthocyanidins is about 20 mg/d, being Cy the most common [68] and in Polish adults participants of the HAPPIEE study, 56% of the daily ACNs intake came from black currant, beans and strawberries [74]. Eastern countries seem to have a higher intake of flavonoids than Americans or Europeans but their ACNs sources appear to be lesser than their isoflavone/proantocyanidin sources. According to KNHANES 2007–2012 [75], the mean daily intake of total flavonoids in Korean adults was 318 mg/d/person, from proanthocyanidins (22.3%), flavonols (20.3%), isoflavones (18.1%), flavan-3-ols (16.2%), anthocyanidins (11.6%), flavanones (11.3%) and flavones (0.3%); major contributing food groups to flavonoid intake were vegetables (20.5%) such as onions (9.6%) and fruits (54.4%) such as apples (21.9%), mandarins (12.5%), grapes (9.0%) and other fruits (1.4%). It should be remembered that the habitual intake of ACNs and Cy3G may vary widely among populations, regions, and seasons and among individuals with different education, financial status and the lack of adequate dietary assessment instruments (e.g., 24FR vs. FFQ) or incompleteness of ACNs food composition tables [76].

In conclusion, dietary choices can have a substantial impact on both the amount of ACNs (and Cy3G) consumed and the associated health effects. In this sense, recent advances in agricultural and food technology have driven the international market of berry fruits at a lower cost. For example, according to the Agri-food and Fisheries Service the production of berries in Mexico has increased almost three-fold in the last years. As a consequence, the intake of ACNs and particularly of Cy3G will steadily increase in the near future.

## 4. Foodomics

**Figure 3:**
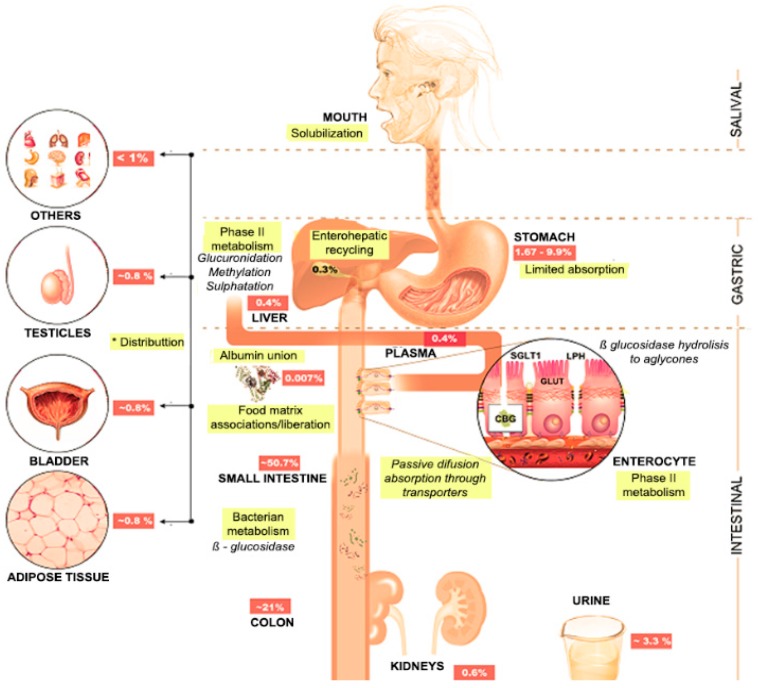
Cy3G metabolic fate in humans. Expressed as percentage of original Cy3G intake. See abbreviations section for the meaning of term.

**Figure 4:**
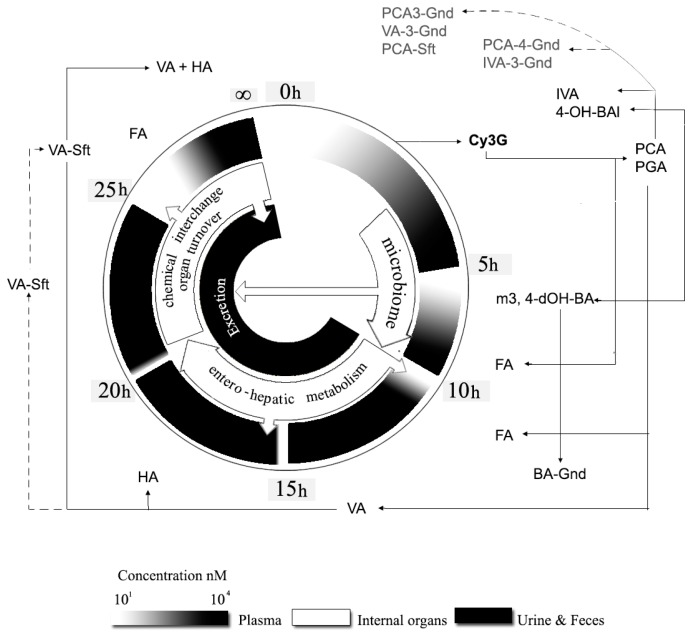
Time-course bioavailability of Cy3G and its main metabolites. Note: The absorption and biotransformation of cyanidin-3-*O*-glucoside (**Cy3G**) into derivates and metabolites are depicted clockwise in this figure and explained in detail within the text. See abbreviations section for the meaning of terms.

According to Capozzi and Bordoni [77], the study of the food domain as a whole to reach an optimized human health and well-being is referred as to *foodomics*. Accordingly, after intake, Cy3G within a food matrix must be subject to different bioprocesses [26] in order to exert its functional action within target organs. It must be releasable (bioaccessible) from its food matrix, presented to and absorbed by gut epithelial cells, transported in the bloodstream, bio-transformed in target tissues and finally excreted in urine and feces [78]. During ADME, Cy3G undergoes many transformations that reduce or enhance its bioactivity such as acid and enzymatic modifications, transport across gut epithelium, phase I and II metabolism and delivering mechanisms to name a few [26]. Particularly, the specific enzymatic action on Cy3G along with its enhanced capacity to be absorbed (as compared to its aglycone) have a great influence on its metabolic fate. A step-by-step review on Cy3G’s metabolic physiological fate (Figure 3) and foodomics (Figure 4) is discussed in detail in the following paragraphs.

### 4.1. Oral Cavity

ACNs-rich edible sources are firstly subject to oral metabolism. The oral pH (6.8 ± 0.2) favors the quinoidal form of Cy while the glucose moiety improves its solubility (Table 1). Many mechanical and chemical factors in the oral cavity lead to the first release of PC (including Cy3G) by changing the texture of foods and dissolving their components [79]. Oral processing of foods involves mastication, lubrication (saliva), enzymatic hydrolysis and epithelial transportation before the bolus is propelled to the esophagus (swallowing). Food composition, structure, rheology and flavor are important determinants of bolus formation [80] and the proper release of ACNs. For example, the oral perception of dryness (astringency) and roughness (puckering) for positively charged emulsions such as ACNs-based beverages is related to their strong interaction with saliva components such as His/Pro-rich proteins [81] causing their precipitation.

As previously mentioned, Cy3G can bind to human proteins by non-covalent forces mainly in acidic conditions [31,32]. Since a high intake of Cy3G (e.g., from berry juices) results in α-amylase inhibition at neutral conditions [82], it is probably the case that Cy3G undergoes a slow sequence of water addition (hemiketal formation) and subsequent C-ring opening (chalcone formation). Here, the concentration and time of exposure are important regulators of Cy3G’ fate. Kamonpatana et al. [83] reported that almost 50% of Cy3G, Cy3Ga, Cy3A and, Cy3X (see Table 1 for nomenclature) were degraded by oral enzymes in a time-dependent manner during 60 min ex vivo incubation at 37 °C with human saliva suggesting a limited impact of type of sugar moiety.

Nevertheless, Cy3G can be bio-transformed in the oral cavity. Mallery et al. [84], in an experiment involving three sequential mouthwashes with a black raspberry solution (BRB; 10% *w*/*v*) rich in Cy3R, Cy3G, Cy3XR and, Cy3Sa (see Table 1 for nomenclature), demonstrated that these molecules are efficiently de-glycosylated by salivary β-glycosidase (microbial derived) and bio transformed to protocatechuic acid (PCA) and Cy-glucoronides, implying an ADME process within the oral cavity; These events are surely related to Cy3G’s preventive action toward smoking-related periodontal diseases [4] or prevention from oral cancer [85]. However, whether these important events are related to the oral transit time or epithelial accumulation of Cy3G at this level needs further investigations.

### 4.2. Stomach

Gastric conditions represent the second barrier for the bioaccessibility and bioavailability of Cy3G (Figure 3). In simulated gastric conditions, Cy3G (as flavylium cation) seems to be stable to acid pH (1.3 ± 0.2) and pepsin action and can be easily released from complex and pH-denatured food matrices [79,86,87]. Since certain studies involving rats and humans indicate that Cy3G reaches plasma very rapidly (0.25–2 h) after intake (Figure 4) [26,27,88], this has led to the conclusion that Cy3G is efficiently absorbed by the gastric epithelia (1%–10% intact, 10%–20% as first-pass metabolites) by active but not by passive diffusion [33,56]. Many authors have suggested active transport mechanisms in gastric epithelial cells, mainly the bilitranslocase transporter [88,89], NA^+^-glucose transporter 1 (SGLT1), glucose transporter 1 (GLUT1) and 3 (GLUT3) and mono-carboxylated transporter 1 (MCT1) [86,87]. The molecular mechanism by which Cy3G crosses the gastric epithelia seems to be related to a specific conformation in its B-ring and glucose moiety with the transporter [30,90]. If that is the case, then it seems likely a pH-dependent competition between d-(+)-glucose and Cy3G [56].

In situ gastric perfusion studies have confirmed this absorption phenomenon but also an extensive first-pass metabolism that reduces Cy3G bioavailability [12,13,56]. In particular, Felgines et al. [28] demonstrated that the percentage of absorption of ACNs in red oranges [Cy3G and Cy-3-malonylglucoside (Cy3MG)] after an intra-gastric injection of about 54 nmol (each) was 21% and 18%, respectively. This finding coincides with the hydrophilic nature of each molecule (Table 1). Ex vivo experiments have confirmed that Cy3G is absorbed and metabolized by human gastric cells (AGS and KATO III) but its anti-proliferative activity (AAP) is lower than that of its aglycone [91,92]. Lastly, as stated earlier, in acidic conditions, many intermolecular binding events can occur between Cy3G and dietary and/or gastric components negatively charged [9,29]. However, whether any other phytochemicals exerts a synergistic or an antagonistic effect on Cy3G’s bioavailability at this level remains obscure.

### 4.3. Small Bowel

The third moment on Cy3G metabolism is within the small bowel. Unlike gastric conditions, the physical and chemical microenvironment in the small intestine reduces Cy3G’s bioavailability by 40–50% (Figure 3). Studies performed in mice [28], humans [14,26] and rats [12,93] confirm this finding. Factors such as pH, Cy3G’s releasability from the food matrix, pancreatic and brush border enzyme action, transportation mechanisms and enterocyte’s phase I/II metabolism, are responsible for the bioavailability of Cy3G, Cy and their metabolites (degradation products or phase II metabolites). At intestinal pH (8.2 ± 0.2) Cy3G returns to its quinoidal form (negatively charged and highly unstable) while its glucose moiety remains neutral. Also, uncertain factors/enzymes perform the further de-glucosylation of Cy3G since lactase-phlorizin hydrolase (LPH; EC 3.2.1.62) or cytosolic β-glucosidases do not seem to participate [12]; however, other authors still consider the action of LPH on Cy3G molecule [94]. Nevertheless, it is noteworthy that the cleavage of its glucose moiety is not a prerequisite for Cy3G chemical breakdown and splanchnic metabolism [93,95] although it is an important mediator of its trans-epithelial transport [27].

Cy3G metabolism at this level largely depends on its ability to release from the food matrix. For example, Kuntz et al. [96] demonstrated in a self-crossover intervention with humans that a grape/blueberry juice (low viscosity) was as good as a smoothie (high viscosity) in terms of ACNs’ bioavailability. Also, Ribnicky et al. [97] used an automated upper GI-simulation system to study the bioaccessibility and ileal recovery of blueberry-ACNs rich extract (500 mg) in the absence (fasting state) and presence of fat (fed state), showing a clear effect of fat over the BA and IE of Cy-glycosides. Also, the inhibitory capacity of Cy3G toward pancreatic enzymes is dose-dependent. For example, Sui et al. [98] performed in vitro and in silico studies to evaluate the inhibitory capacity of several ACNs against porcine pancreatic α-amylase, showing the following trend: Cy3G (Ki = 0.014 mM) > CyR > Cy3, 5GG > Pn3G (Ki = 0.045 mM). Lastly, Akkarachiyasit et al. [99] showed that Cy3Ga and Cy3G are potent inhibitors of sucrase and α-amylase with IC_50_ values of 0.50 ± 0.05 and 0.30 ± 0.01 mM.

Many aspects of Cy3G’s transport mechanisms have been learned from the use of Caco-2 cells as an absorption model. Most of these studies suggest that, unlike other flavonoids, ACNs could be transported in intact aglycone forms from berries (and their products) except for black currant and some grape varieties [22]. Kuntz et al. [96] evaluated the uptake of ACNs from a grape/blueberry extract (with 63 mg/L of Cy3G) in Caco-2 (ATCCqHTB37e) monolayers showing that Cy3G, although not being the major ACN, has a higher absorption efficiency when compared to Pt3G (103 mg/L) or Dp3G (96.4 mg/L) but this efficiency was better at acidic but not neutral pH. Zou et al. [90] showed that the transport of Cy3G from apical to basolateral is mediated mainly by glucose-transporters (SGLT1 and GLUT2) but a “saturation” effect (up to 40 μM) during Cy3G active transport was observed. Lastly, the presence of other food components has been shown to have a major impact on ACNs transport [22]. In particular, the presence of phospholipids and terpenes enhances Cy3G and Cy3R absorption in Caco-2 cell monolayers [100], contrary to what was found by Ribnicky et al. [97] using TIM-1.

On the other hand, Hassimotto et al. [56] using jejunal everted sacs from rats demonstrated that Cy3G is efficiently transported by SGLT1 in a dose dependent way but Cy is not. However, in the mouse small intestine, Cy3G absorption not only depends on SGLT1 transporter but there might be an exclusive transporting mechanism for flavonoid-like molecules since quercetin-3-glucose seems to inhibit Cy3G absorption [101]. At this point, it should be mentioned that there is not much evidence on the para-cellular transport of Cy since it is more hydrophobic than Cy3G (Table 1). Lastly, luminal Cy3G’s metabolites are efficiently absorbed at intestinal level due the contribution of those derived from entero-hepatic (EHM; Figure 4) or from oral metabolisms [12,13,84].

Once inside the enterocyte, Cy and Cy3G could be either transformed to other PC (particular phenolic acids) and derivatives in phase I metabolism or to several conjugates (methylated, glucuronidated or sulphated) in phase II metabolism. Microbial but not host metabolic machinery is responsible for producing phase I metabolites, although there is a controversy if this event takes place in the small bowel or it is also a result of EHM. In phase II metabolism, many enzymes such as phenyl sulfotransferases (PST), uridine 5′diphosphate glucuronosyltransferases (UGT) and catechol*-O-*methyltransferase (COMT) activities can modify the Cy (and other anthocyanidins) structure making it more water-soluble and facilitating their further elimination by the kidneys [12,13,28,93].

PCA and PGA have been consistently reported as the main phase I Cy-derived metabolites/degradants [23,27]. Cy and PGA are more hydrophobic molecules than PCA and so, they can passively diffuse through biological membranes, reaching the plasma in the first 2 h (Figure 4). Another reported reaction, although it is not clear where exactly it happens, involves Cy (or Cy3G) methylation to produce Pn (or Pn3G) both having almost the same in vivo bioactivity [9,14]. Recently, Fang [13] proposed the major pathways for Cy3G metabolism in liver microsomes: after deglycosylation, Cy produces PGA from its A-ring, ferulic acid (FA), 3,4-dihydroxyphenyl acetic and 4-hydroxyphenylacetic acids from its B-ring and 3,4-dihydroxybenzaldehyde (PCA immediate precursor) also from its B-ring.

Lastly, PCA undergoes several structural transformations by certain phase I enzymes to produce hippuric (HA), vanillic (VA) or isovanillic (IVA) acids. Cy3G, Cy, PCA, VA and IVA are further metabolized to their specific glucuronide or sulfate conjugates by phase II enzymes. According to Ferrars et al. [14], the *t*_max_ for both Cy3G and Cy3-glucoronide is 16 times lower (1.8 h) than that observed for VA-sulphate (30.1 h) while *C*_max_ for PCA-3-*O*-glucuronide is 177 times lower (11 nM) than for HA (1,962 nM). Lastly, most of these metabolites have been found in rats but they also produce β-resorcylic acid (βRA: 2,4-dihydroxybenzoic acid) [102] and other methylated derivates [93].

### 4.4. Large Bowel

Cy3G and derived metabolites that surpassed absorption from the small bowel can be finally released from fibrous food matrices (also known as macromolecular antioxidants), transformed by the microbiome [102] and then absorbed by colonocytes. The large bowel contributes to the remaining deglucosylation, phenolic acid production and phase II conjugation events and so less than 0.005% of Cy3G is excreted intact [14]. As occurred in the small bowel, the C-ring rupture and the Cy chalcone formation [103] leads to the apparition of molecules derived from hydroxybenzoic (OH-BA) and phenylacetic acids, VA, IVA, FA and HA (Figure 4) which can be further eliminated in feces and urine (by means of EHM). These metabolic transformations are favored by the slightly basic pH present at this level [79] where Cy3G and Cy are highly unstable. However, certain metabolites such as 2-OH-4-methoxybenzoic acid, 4-methoxybenzaldehyde, methyl-VA and caffeic acid are specifically produced within the large bowel [14].

Hanske et al. [102] evaluated the impact of human intestinal bacteria on the fate of Cy3G in a rat model as compared to germ-free (GF) rats. HMA rats excreted 3× and 2× more phase I (unconjugated: OH-BA derivates) and phase II (conjugated) Cy3G metabolites than GF rats. Also, Pn and 3-OH-cinnamic acid were excreted in urine from HMA but not GF rats. Also, during microbial fermentation of purple sweet potato (a rich source of Cy-mono and di-glycosides, and Cy3G), produces a substantial amount of short-chain- and lactic acids and partially fragmented to phenolic acids. Whether microbial populations are benefited by the dietary fiber, phenolic compounds or both in a synergistic or specific interaction is currently under investigation [103]. Ozdal et al. [104] recently reviewed the state of the art in this area.

### 4.5. Splanchnic Metabolism

Isotope tracer studies have been very useful to establish the extent to which Cy3G is metabolized by splanchnic organs [94,105]. Czank et al. [26] evaluated the post-prandial kinetics of ^13^C_5_-Cy3G (500 mg) after intake. The relative ^13^C_5_ bioavailability was 12.4% (5.4% urine, 7.0% breath) and the percentage of recovery in feces, breath and urine was 44%. Cy3G metabolites peaked (*C*_max_ = 6 μM) at 10.3 h but their half-life ranged from about 12.4 to 51.6 h. Despite that ^13^C elimination was faster by urine (0–1 h, 90.3 μg/h) than for breath (6 h, 132.9 μg/h) or feces (6–24 h, 557.3 μg/h), the highest concentration of ^13^C was recovered in feces (43.2 μM) and urine (10.8 μM), respectively. The authors concluded that Cy3G (and its metabolites) was as bioavailable as flavan-3-ols and flavones (2.5%–18.5%) but their HPLC-MS/MS method failed to detect other diverse breakdown products and metabolites (56%).

De Ferrars et al. [14], using HPLC-ESI-MS/MS and non-compartmental pharmacokinetic modelling, reported the metabolic fate of Cy3G using the same oral dose and tracer as Czank et al. [26]. Plasma pharmacokinetic parameters for ^13^C_5_-Cy3G were: maximal concentration *C*_max_ = 141 ± 70 nM, *t*_max_ = 1.8 ± 0.2 h, *t*_1/2_ = 0.4 h and, area under the curve (AUC)_0–48h_ = 279 ± 170 mM. For metabolic products, *C*_max_, *t*_max_ and *t*_1/2_ ranged from 10 to 2000 nM, 2 to 30 h and 0.5 to 96 h, respectively. Moreover, the abundance of ^13^C-metabolites was 42 times higher than ^13^C_5_-Cy3G at their respective maximum serum concentrations. The authors confirmed the relative bioavailability of Cy3G reported by Czank et al. [26] but also identified 35 important metabolites of which 17, 31 and 28 of them were identified in plasma, urine and feces, respectively [13] which indirectly sustain the aforementioned EHM of Cy3G’s metabolites. Also, typical urinary recoveries of ACNs are <2% of the intake [88] but for Cy3G seems to be much lower (0.024%) [14], although that of its metabolites is not accounting for about 3.0%. In fact, metabolites such as Cy-glucoronides and Pn3G are only found in urine.

Figure 4 attempts to integrate the most recent findings on Cy3G’s foodomics but also postulates new questions for further research. For example, ADME studies (acute consumption) provide enough evidence to ensure that Cy3G has a very short half-life in plasma since it is rapidly metabolized during absorption and EHM [14]. However, regular consumption of ACNs-rich foods could results in a different molecular turnover than that observed after acute consumption. In support of this idea, Khymenets et al. [106] recently reported that a sustained consumption (human volunteers) of a functional beverage based on grape skin extract increased the number of molecular species derived from microbial metabolism of flavan-3-oles, as compared to acute consumption. Also, the metabolic integration of many organs with specific antioxidants needs could result in a different fate for Cy. Lastly, Cy3G’s metabolites can be deposited in hydrophobic (e.g., adipose tissue, liver, testicles) or hydrophilic (e.g., bladder, kidneys) tissues and so long-lasting metabolites such as VA, HA and FA may suffer chemical interchange reactions upon their entry into these tissues. For instance, de Ferrars et al. [14] indicated that urine contains trace amounts of Pn3G which strictly come from the methylation of Cy3G only in renal tissues. Also, more than 100 and 700 organic acids and derivates and aromatic heteropolyciclic compounds have been identified in the human urine metabolome including HA (HMDB00714), OH-HA (HMDB13678), *trans*-FA (HMDB00954), benzoic acid (BA; HMD01870), 4-OH-BA (HMDB13678), VA-lactic acid (HMDB00913) and VA-mandelic acid (HMBD00291), which are commonly found as breakdown products from a wide range of foods, drinks, drugs, environmental contaminants, endogenous metabolites and bacterial by-products [107]. Nevertheless, more in vitro studies on the specific effects of the physical-chemical (e.g., pH, ionic strength) and biochemical (e.g., phase I and II enzymatic modifications) changes in Cy3G combined with in vivo or ex vivo studies are needed in order to elucidate many important physiological implications for the bioactivity of this molecule.

## 5. Cy3G Health Effects

Generally speaking, anthocyanins seem to be non-toxic molecules within the margin of a normal consumption. Their safe intake (LD_50_) has been set up to 25,000 mg in mice and 20,000 in rats per kg body weight with no apparent adverse effects. Rabbits orally fed with ACNs (6 g/kg bw) had no pressure changes while, in guinea pigs and dogs, no sub-chronic toxic effects were observed (3 g/kg bw) [108]. Moreover, Charoensin et al. [109] provide information concerning the safety and anti-mutagenic potency of Cy3G. On the other hand, and despite their apparent chemical instability and low bioavailability, ACNs-rich foods have demonstrated their biological efficacy in a wide range of human diseases (acute and chronic) and the molecular mechanisms by which they exert their benefits have recently been reviewed by Li et al. [15]. However, a critical and thorough analysis of this evidence provides little insight into the specific role of Cy3G. Nevertheless, epidemiological studies, experimental trials using laboratory animals, in vitro assays using cell lines and ex vivo tissues and clinical studies with humans all together provide enough metabolomic evidence on Cy3G’s bioactivity and nutraceutical potential. In this last part, some of the most recognized health benefits of Cy3G (and metabolites) are systematically evaluated with a special emphasis on metabolomic responses.

### 5.1. Human Studies

The first evidence comes from very few epidemiological studies. The wide range of ACN intakes from dietary sources observed in many population-based surveys has been related to certain benefits against NCCD. Cassidy et al. [110] observed that a higher ACN intake (mostly coming from apples, pears, red wine, and strawberries) was inversely associated with many inflammatory biomarkers. Mehta et al. [111] demonstrated that consuming ≥2 cups/week of blueberries (or strawberry to a lesser extend) was associated with a slower rate of annual decline in lung function but no significant associations were observed for red wine intake. Also, Ponzo et al. [112] reported that a higher anthocyanidin intake is associated to a lower incidence of CVD events and all-cause mortality. Although we cannot ensure a priori other health benefits, these studies demonstrate that in vivo RSC of ACNs could be the main mechanism of action. At this point, we must remember that regular consumption of berry fruits makes important contributions to total ACNs and Cy3G daily consumption since the latter is the most common ACN (Table 3).

Clinical studies in humans have provided additional pieces of information on Cy3G’s bioactivity. For example, Cy3G has benefits for cardiovascular health. Hassellund and others [113] performed a randomized and double-blind crossover study [placebo vs. 640 mg/d/4 weeks (purified ACNs supplement)] showing that several ACNs peaked within 1–3 h but so did HDL-cholesterol, while Davinelli and others [114] using the same protocol but different ACN sources [placebo vs. 486 mg/d/4 weeks (Delphinol^®^ from maqui berry, Huechuruba, Santiago, Chile] observed a decrement in LDLox and F2-isoprostanes levels but both studies did not show any other apparent benefit for CVD markers. According to this, Zhu et al. [115] showed significant changes in serum HDL- (up) and LDL- (down) cholesterol as well as high sensitivity C-reactive protein (hsCRP) and soluble vascular cell adhesion molecule-1 (sVCAM-1) levels after the 24-week ACNs supplementation. The same results have been reported with ACNs from strawberry, modulating these inflammatory biomarkers and indirectly improving insulin action [116]. It is noteworthy that endothelial health is related to the regulation of nitric oxide production, and the latter could be modified by several types of flavonoids including ACNs [117,118]. As to Cy3G’s anticancer activity, Mallery and others [84] confirmed that Cy derivatives (including Cy3G) from black raspberries (BRB) are extensively metabolized and retained within the oral cavity of healthy humans and Knobloch and others [85] showed that providing oral troches of freeze-dried BRB (FD-BRB) to patients with oral squamous cell carcinomas (OSCCs) for 14 day enhances the expression of pro-survival genes (*AURKA*, *BIRC5*, *EGFR*) and reduces other pro-inflammatory genes (*NFKB1*, *PTGS2*). Moreover, the acute intake of a blueberry dry extract regulates DNA methylation in patients with colorectal adenocarcinomas, despite the inter-individual variability [119]. Most if not all of these metabolic effects are related to Cy’s or Cy3G’s binding capacity toward different macromolecules including key proteins and enzymes, some of which will be discussed in Section 5.3.

### 5.2. Cell Lines and Rodent Models

Most findings regarding Cy3G’s bioactivity come from in vitro cell-based assays (Table 4) and in vivo experimental studies with rodents (Table 5). Cellular and rodent models provide concurrent and complementary evidence in many cases. Cy3G’s bioactivities include most commonly DNA-RSC, gastro-protective, anti-inflammatory, anti-thrombotic, insulinotropic, anti-microbial, chemopreventive and epigenetic effects. In turn, these actions may help to prevent *H. pylory* infection, periodontal and age-related diseases, type 2 diabetes mellitus, CVD, metabolic syndrome and oral cancer, to name just a few [3,4,5,6,120,121,122,123,124], involving specific molecular mechanisms (for both Cy and Cy3G, either pure or within plant food matrices) including RSC, epigenetic action, protein binding capacity and enzyme inhibition and many other mechanisms [125,126,127,128,129,130,131,132,133,134,135,136,137,138,139,140,141,142,143,144,145,146,147,148,149] some of which are discussed in the following paragraphs.

The RSC and molecular competition ability of Cy3G (and Cy) may help to prevent certain inflammatory processes, CVD, aging and cancer [10]. For example, senescent and cancer cells are also susceptible to DNA cleavage due to environmental stressors which generate free radicals and activate oxidative enzymes such as xanthine oxidase that can be attenuated by Cy and Cy3G [120]. Also, results from animal studies suggest that Cy3G may slow or inhibit the absorption of lipids and glucose in the intestine [15,100,121,122] confirming the postulated mechanisms in cells [1,118,119]) and the physiological impact in humans [113]. Also, Ziberna et al. [125] indicate that Cy3G provides protection against oxidative stress-related CVD since it is transported in EA.hy926 cells by a specific bilitranslocase and accumulates in the vascular endothelium exerting anti-ischemic activity in the isolated rat heart. A special mention should be made of the metabolic bioactivity of Cy3G in adipose cells, both in vivo and in vitro. Tsuda et al. [126] showed that Cy3G and Cy both up-regulate human adiponectin, uncoupling protein-2, acylCoA oxidase-1 and perilipin but down-regulate plasminogen activator inhibitor-1 and IL-6. Björk et al. [127] observed that exposing white adipose cells to the omega-3-fatty acid docosahexaenoic acid + Cy3G suppresses the secretion of interleukin-6 and monocyte chemoattractant protein-1 (MCP-1/CCL2) and decreases its basal lipolytic activity. However, Tsuda et al. [128] found that Cy3G and Cy up-regulates the hormone-sensitive lipase gene and enhances the lipolytic activity of rat adipocytes. These findings suggest a species-specific effect of Cy and Cy3G.

However, the biological effects demonstrated in laboratory animals and in vitro assays could be overstated. In many cases, the amount required to achieve a specific biological action in general is much larger than that obtained from dietary sources. For example, the average amount of Cy3G employed in rat/mice bioassays far exceeds (about 30–60 times) the amount that can be obtained from a single dietary source (Table 3) [132], although in many cases the amount derived from an habitual diet is sufficient to achieve certain benefits. Moreover, as stated in previous sections, Cy3G from natural sources or manufactured nutraceuticals is largely limited by its splanchnic metabolism and so its fate in target internal tissues is limited. In this case, entrapping agents such as malto/cyclodextrins or liposomes are convenient alternatives to preserve its bioaccessibility and bioavailability within the GI tract. In this sense, concentrated sources of Cy3G such as purees or freeze-dried fruits could result not only in a much higher intake of Cy3G but also constitute a way to protect it from GI harsh conditions [87,129]. Other bioactivities related to Cy3G’s RSC, molecular competition and epigenetic action are shown in Table 4 and Table 5 [133,134,135,136,137,138,139,140,141,142,143,144,145,146,147,148,149].

### 5.3. Physiologically-Relevant Molecular Mechanisms

The aforementioned bioactivities of Cy3G, its aglycone (Cy) and derived metabolites rely mostly on the following mechanisms: RSC, epigenetic action, competitive protein-binding and enzyme inhibition. The unspecific RSC mechanism of these molecules has been extensively discussed in Section 2 as related to their structural features [130,131], while their epigenetic mechanisms have been postulated in almost all studies involving cancer cell lines and tumor xenografts. On the latter, important epigenetic modifications known to regulate gene expression are exerted by many flavonoids such as naringenin, kaempferol and quercetin [150] and the combination of flavonoids and chemotherapy seems to be an interesting approach for cancer treatment [151]. However, molecular studies involving Cy3G or Cy as epigenetic effectors are still scarce.

As to their macromolecular-binding and enzyme inhibition capacities, the first body of evidence comes from all in vitro and in silico studies using pure DNA (152) or protein (31–32, 153–156) systems. Zhang et al. [152] studied the binding capacity of Cy and Cy3G to calf thymus DNA demonstrating a Δ*λ* to the ultraviolet-visible spectra of this nucleic acid, indicating the formation of the DNA-Cy and DNA-Cy3G complexes with an intercalative binding mode evidenced in their fluorescence spectra and that Cy3G binds to DNA more efficiently than Cy. Both molecules also bind salivary [81,83,84] and blood [31,32] proteins, a fact that may modify their fate within GI and their bloodstream transport. Spectroscopic studies suggest that Cy3G spontaneously binds albumins by means of weak forces such as hydrogen bonds and Van der Waals forces and hydrophobic interaction on a minor scale [31,32]. Cy3G binds to BSA in its IIA sub domain and it is surrounded by key hydrophobic and non-polar (Ala, Leu, Phe, Gly, Trp and Tyr) and polar (Arg, Asp, Glu and Lys) residues within the hydrophobic cavity of site II’ [153]. The same phenomenon has been observed in HAS, but also certain structural features of both anthocyanidins/ACNs, modify their binding capacity toward HAS: At pH 7.0 (high) and pH 4.0 (low), there is a differential electrostatic environment affecting the binding capacity of ACNs in their quinoidal form [154,155]. Also, the binding constant of Cy3G has been reported to be higher for myoglobin than for BSA hemoglobin, structurally associated to its binding capacity toward α-helices [32]. Lastly, evidence on the differential binding and inhibitory capacity of Cy and Cy3G toward GI enzymes has been disentangled. Akkarachiyasit et al. [99] revealed that Cy3G is a much stronger inhibitor of both intestinal α-glucosidase and pancreatic α-amylase as compared to Cy. While Adisakwattana et al. [156] found that glucose substitution at 3-*O* (increases) and 5-*O* (reduces) positions in Cy, it modifies its inhibitory activity toward α-glucosidase.

**Figure 5:**
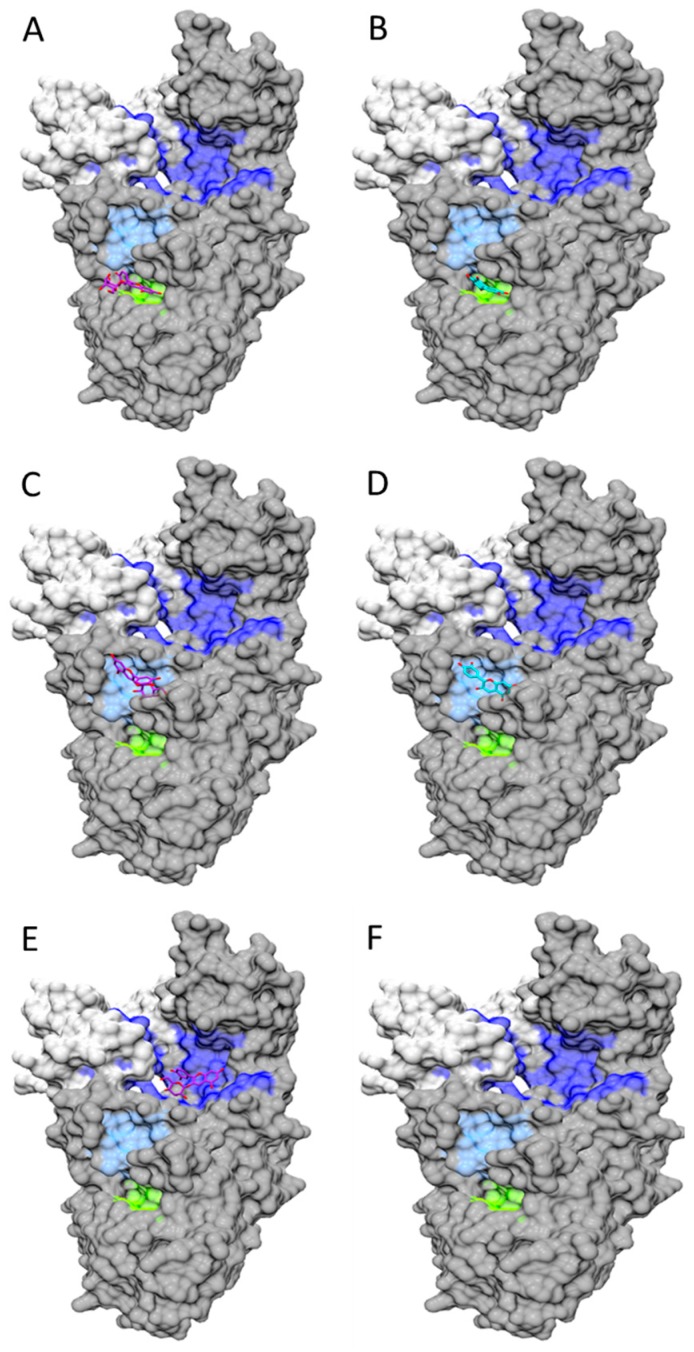
Docking simulation of Cy3G and Cy toward porcine lipase/colipase complex. Docking simulation details are described in Section 5.3. Cy3G (**left**) and Cy (**right**) within (**A**,**B, light green**) and near (**C**,**D; light blue**) the active site of lipase (**dark grey**) and at the binding region with colipase (**intense blue**; **E**,**F**).

**Figure 6:**
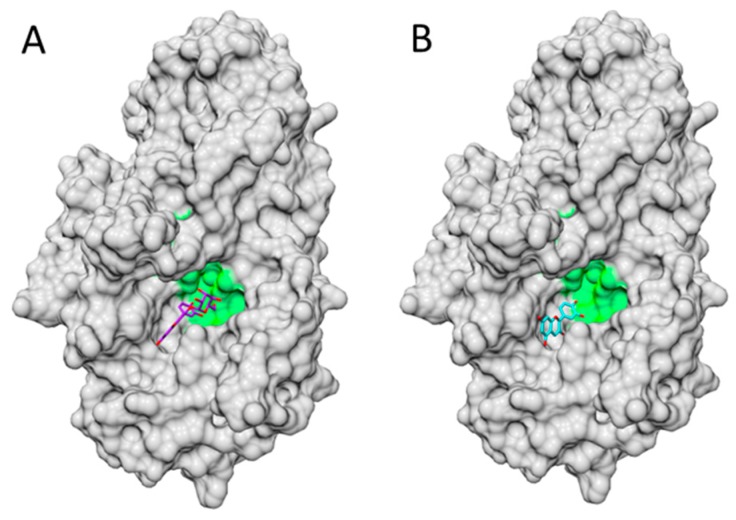
Docking simulation of Cy3G and Cy toward porcine amylase. Docking simulation details are described in Section 5.3. Cy3G (**A**); Cy (**B**) within enzyme’s (**light grey**) active site (**green**).

Molecular docking simulations have provided further evidence on the structural features and mechanisms involved in Cy3G’s and Cy’s binding capacity toward different proteins. Oliveira et al. [86] studied the binding capacity of Cy, Dp and Mv glucosides toward hGLUT1 showing that the pattern of hydroxylation determines their hydrogen-bonding with this protein at ΔG_binding_ = −9.07, −8.13 and −7.36 kcal/mol, respectively [86] while Perez-Diaz et al. [157] showed the ability of four glutathione *S-*transferases (H-site, N-terminal domain) from *Vitis vinifera* to bind and even transport Cy3G (ΔG_binding_ = −2.43 to −2.03). However, similar studies comparing Cy’s and Cy3G’s differential binding capacity toward GI enzymes have not been reported yet. Docking simulations [flexible ligand/rigid enzyme (100 × 100 × 100 grid points) docking] using AutoDock Vina [158] and UCSF Chimera [159] packages, Cy3G (PubChem: 92131208) and Cy (PubChem: 128861) as ligands and porcine lipase/colipase complex (RCSB PDB-1ETH) and porcine amylase (RCSB PDB-1PIF) as templates are depicted in Figure 5 and Figure 6. Cy3G and Cy have three potential binding sites for porcine lipase–colipase complex (Figure 5) within (A, B) and near (C, D) the active site and at the binding region with colipase (E, F). While Cy presumably binds more effectively than Cy3G within (ΔG_binding_ = −9.8 vs. −7.0 kcal/mol) and near (ΔG_binding_ = −8.8 vs. −8.0 kcal/mol) the active site, an inverse phenomenon is observed at the lipase-colipase region (ΔG_binding_ = 0.0 vs. −7.7 kcal/mol). According to Figure 6, Cy also binds more strongly to amylase when compared to Cy3G (ΔG_binding_ = −8.9 vs. −7.8 kcal/mol). Based on this data, a different lipase-colipase and amylase inhibitory activity could be expected and possibly a different hypolipidemic and hypoglycemic effect in clinical studies. However, these statements should be proven in future studies.

## 6. Future Prospects

Cy3G is a fascinating PC in many ways. For this reason, it has been studied in many disciplines including chemical, agricultural and biomedical sciences. Unlike other ACNs, its chemical nature has a profound effect on its in vitro and in vivo behavior, sometimes improving or otherwise limiting its antioxidant capacity and stability, bioaccessibility and bioavailability in the gastrointestinal tract and its nutraceutical effects in living systems. However, an adequate and sustained consumption of Cy3G-rich sources such as certain berry-based juices, purees or concentrates could contribute to maintaining an adequate amount of metabolites in plasma and certain tissues to ensure its nutraceutical effects. However, the Cy3G puzzle is not solved yet since more foodomic studies in plants, animals and man are required to discover new chemical and biological aspects of ACNs widely distributed in nature and their therapeutic effects in people with serious illnesses. Lastly, more studies on the assessment of Cy and Cy3G in equilibrium (bound/unbound) toward physiologically relevant macromolecules are needed in order to understand their physiological relevance

## Figures and Tables

**Table 1:** Mass spectral data and physicochemical characteristics of Cyanidin (Cy) and glycoside-derivates ^1^.

Code	Name	[M]^+^ (*m*/*z*)	MS/MS (*m*/*z*)	Solubility (mg/mL)	LogP	Å^2^
Cy	Cyanidin (anthocyanidin)		287	0.049	3.05	114.3
Cy3A	Cy-3-arabinoside (pentose)	419	287	0.41	1.06	173.21
Cy3X	Cy-3-xyloside	419	287	0.41	1.06	173.2
Cy3G	Cy-3-glucoside	449	287	0.6	0.39	193.4
Cy3Ga	Cy-3-galactoside	449	287	--	0.24	193.4
Cy3Aga	Cy-3-(6′′-acetyl)-galactoside	491	287	0.39	0.82	199.5
Cy3Sa	Cy-3-sambubioside	518	287	--	--	--
Cy3,3′′MG	Cy-3-(3′′-malonyl)-glucoside	535	287	0.47	0.68	236.8
Cy3,6′′MG	Cy-3-(6′′-malonyl)-glucoside	535	449/287	0.45	0.68	236.8
Cy3Sa	Cy-3-sambubioside	581	287	1.17	-1.1	252.4
Cy3dOXG	Cy-3-(dioxaloyl)-glucoside	593	287	0.17	2.55	280.2
Cy3R	Cy-3-rutinoside	595	449/287	0.9	−1.64	252.4
Cy3XR	Cy-3-xylosylrutinoside	727	581/287	2.52	−2.1	311.3
Cy3GR	Cy-3-glucosylrutinoside	757	287/611	3.85	−2.8	331.5
Cy3,5GG	Cy-3,5-diglucoside	611	449/287	--	−2.3	272.6
Cy3So	Cy-3-sophoroside	611	287	--	--	260
Cy3Sa5R	Cy-3-sambubioside-5-rhamnoside	727	581/433/287	--	--	--
Cy3So5R	Cy-3-sophoroside-5-rhamnoside	757	611/433/287	--	--	--

^1^ See abbreviations section for non-defined terms.

**Table 2:** Voltammetric oxidation of and Cy3G and its aglycone ^1^.

Molecule	pH	Epa/mV	Technique	Electrodes	Ref.
Cy3G	3.5	490, 980	DPV	WE: Glassy carbon RE: Ag|AgCl	[47]
	4.5	420, 815			
	7.0	310, 500			
Cy3G	2.2	548	CV	WE: Glassy carbon RE: Ag|AgCl	[52]
	4.8	400			
	5.9	310			
	6.9	230			
Cy3G	2.0	500	ASSWV	WE: Paraffin rod impregnated RE: Ag|AgCl	[52]
Cy		403			
Cy3G	1.0	617	CV	WE: Platinum RE: SCE	[53]

^1^ See abbreviations section for the meaning of each term.

**Table 3:** Cyanidin-3-*O*-glucoside (Cy3G) content in selected edible sources ^1^.

Group	Fruit/Vegetable	Cy3G ^2^	Major ACNs ^3^
Fruits/Berries	Black elderberry	794.13	Cy3G
	Blackberry raw	138.72	Cy3G
	Black Aestivalis grape	18.72	Cy3G
	Gooseberry	2.95	Cy3G
	Nectarine peeled	0.56	Cy3G
	Peach peeled	0.28	Cy3G
	Blackcurrant raw	25.07	Dp3R (304.91)
	Black chokeberry	19.64	Cy3A (252.76)
	Blueberry	14.2	Dp3G (22.6)
	Sweet cherry raw	18.73	Cy3R (143.27)
	Red raspberry	14.89	Cy3So (37.61)
	Raspberry pomace (dry)	41.52	Cy3So (100.1)
	Plum fresh	8.63	Cy3R (33.85)
	Lowbush blueberry	7.5	Mv3G (26.06)
	Redcurrant	3.37	Cy3XR (11.22)
	Strawberry	2.88	Pg3G (47.1)
	Lingonberry	1.42	Cy3Ga (48.69)
	Highbush blueberry	1.37	Dp3Ga (20.50)
	Sour cherry	1.12	Cy3GR (43.63)
	Black grape	1.08	Mv3G (39.23)
	American Cranberry	0.74	Pn3Ga (22.02)
	Cloudberry	0.62	Cy3R (1.86)
Juices/wine	Pomegranate pure juice	3.43	Cy3G
	Blood orange pure juice	1.41	Cy3MG (1.76)
	Red wine	0.21	Mv3G (9.97)
Cereals/legumes	Black bean raw	3.99	Dp3G (20.50)
	Blue maize hybrid	2.25	Cy3G
Vegetables	Black Olive raw	10.62	Cy3R (72.35)
	Red lettuce raw	0.62	Cy3MG (2.91)

^1^ See Table 1 and abbreviations section for the meaning of term ^2^ Average content (mg per 100 mL or 100 g FW), ^3^ (average content).

**Table 4:** Cy3G associated effects-cell lines.

Cell Line	Cy3G dose	Mechanism	Ref.
Erythrocytes	10–100 μM	↓ cholesterol and TBAR in cell membranes	[1]
Human adherent macrophages (from U937 cells), oral epithelial cells (GMSM-K) and gingival fibroblasts (HGF-1)	5–25 μg/mL	↓ IL-6 level (macrophages), cytoprotection (GMSM-K, HGF-1) against nicotine toxicity	[4]
Colon (Caco2), liver (HepG2), prostate (PC3)	Blue maize ACNs (189–500 μg/g)-extract	↓ cell proliferation	[36]
Gastric cancer (KATO III)	12.5 μM	↓ Helicobacter pylori VacA-induced cell death	[91]
Adipocytes (3T3-L1)	50 μM	↓ FoxO1-mediated transcription of lipase	[123]
Hepatome (HepG2)	1–100 μM	↑ fatty acid oxidation and AMPK activity	[124]
Adipocyte	0.5–50 μM + docosahexanoic acid	↓ basal lipolysis , inflammatory markers	[127]
Breast cancer (BT474m MD-MB231, MCF7)	10 μM	↓ invasion / increased expression of ErB2	[133]
Murine thymoma (EL-4T)	2.5–5.0 μg/mL	↓ Il-3 & IL-4 by GATA-3 inhibition	[134]
Pheochromocytoma (PC-12)	IC50, 15.3 μg/mL	↓ ATP-induced [Ca^2+^] increase	[135]
Colon cancer (HT-29)	25 μM	↓ IL-8, nitrite, PGE2	[136]
human aortic epithelial cells	0.5–50 μM	↑ oxiesterol efflux, ↑ABCG1/ABCA1 expression	[137]
Heart (isolated mitochondria)	20 Μm	↑ phosphorylation, ATP production, ↑e^−^ carrier	[138]
Adipocytes (steam cells)	100 μg/mL	↓ IL-6 level	[139]
Ovarian cancer (HO-8910PM)	IC50, 13.8 μg/mL	↑ apoptosis, ↓mucin 4 expression	[140]

**Table 5:** Cy3G associated effects-rodent models.

Model	Protocol (Dose)	Effects	Ref.
Mice (nude), SKH-1	Oral	↓ lipid per oxidation ↑ Glutathione	[3]
Mice, C57BL/6	Oral, 24 h before, (2 mg/kg)	↓ Neuronal apoptosis reducing factor, superoxide level, infarct size	[5]
Mice (obese), C57BL/6	Oral, 5 w, 0.02% diet, (*ad libitum*)	Antidiabetic by modulating c-jun N-terminal kinase	[122]
Mice, apoE (-)	Oral, 12 w, 0.06% diet, (*ad libitum*)	↓ expression of hepatic cholesterol 7a-hydroxylase	[137]
Mice, ovarian cancer	Oral, 2 w, (5 mg/kg)	↓ Growth of ovarian xenograft tumors	[140]
Mice, C57BL/6	Oral, 12 w, (40–200 mg/kg)	↓ weigh gain, insulin resistance, adiposity, leptin	[141]
Mice, apoE (-)	Oral, 8 w, 0.2% diet (*ad libitum*)	↓ atherogenesis, ↑endothelial repair	[142]
Mice, with peritonitis/edema	Oral, 30–60 min before (40 mg/kg)	↓ inflammation, expression COX-2/PGE2	[143]
Mice, diabetic	Oral, 4 w, (300 µg/10g)	↓ blood glucose	[144]
Mice, KK-Ay	Oral	↓ visceral fat ↑ lipoprotein lipase	[145]
Mice, C57BL/6 w/acute alcohol-induced liver injury	Oral, 24–48 h, (10 mg/kg)	↓ plasma IL-6 ,TNF-α, ALT and AST and ↑ SIRT1 p-c-Jun and Bax expression	[146]
Rats (retinal degeneration)	Oral, 5 w, 100 mg/kg	↓ Loss of photoreceptors	[147]
Mice (fetus)	Intra-peritoneal 10–30 mg/kg	↓Neuronal damage by caspase 3 inhibition	[148]
Rats (β-amyloidosis)	Oral, 30 day, 10 mg/kg	↓ cognitive impairment induced by Aβ via the modulation of GSK-3β/tau	[149]

## Abbreviations

The following abbreviations are used in this manuscript:

Δ*A*	Hyperchromic effect
Δ*λ*	Bathochromic shift
ADME	Absorption, distribution, metabolism and excretion
ANR	Anthocyanidin reductase
ANS	Anthocyanidin synthase
ACNs	Anthocyanins
Å^2^	Polar surface area
AOX	Antioxidants
ASSWV	Abrasive stripping square wave voltammetry
βRA	β-Resorcylic acid
BA	Benzoic acid-4-glucoronide
BA-Gnd	Benzoic acid-4-glucoronide
BRB	Black raspberry
BSA	Bovine serum albumin
CHI	Chalcone isomerase
CHS	Chalcone synthase
COMT	Catechol-*O*-methyltransferase
CON	Unknown condensing enzyme(s)
CT	Condensed tannins
CV	Cyclic voltammetry
CVD	Cardiovascular disease
Cy	Cyanidin
Cy3G	Cyanidin-3-*O*-glucoside
DFR	Dihydroflavonol-4-reductase
Dp	Delphinidin
Dp3G	Delphinidin-3-*O*-glucoside
Dp3Ga	Delphinidin-3-*O*-galactoside
Dp3R	Delphinidin-3-*O*-rutinoside
DPV	Differential of pulses voltammetry
EHM	Entero-hepatic metabolism
Epa	Anodic peak potential
F3,5H	Flavanone-3,5-hidroxylase
F3H	Flavanone-3-hidroxylase
FA	Ferulic acid
FLS	Flavonol synthase
FNS	Flavone synthase
GI	Gastrointestinal
GLUT1,2,3	Glucose transporter 1,2,3
HA	Hippuric acid
HAS	Human serum albumin
Hb	Hemoglobin
IVA	Isovainillinic acid
IVA-3-Gnd	IVA-3-glucoronide
LAR	Leucoanthocyanidin reductase
LDOW	Leucoanthocyanidin dioxigenase
LogP	Octanol/water partition coefficient
LPH	Lactase-phlorizin hydrolase
Mb	Myoglobin
MCP-1/CCL2	Monocyte chemoattractant protein-1
MCT1	Mono-carboxylated transporter 1
Mv	Malvidin
Mv3G	Malvidin-3-*O*-glucoside
MW	Molecular weight
NCCD	Non-communicable chronic diseases
OMT	Malvidin (Mv), *O*-methyltransferase
Pas	Proanthocyanidins
PC	Phenolic compounds
PCA	Protocatechuic acid
PCA-3-Gnd	PCA-3-glucoronide
PCA-3-Gnd	PCA-4-glucoronide
PCA-Sft	PCA-sulfate
Pg	Pelargoddin
PGA	Phloroglucinaldehyde
PgANS	Pelargodin specific-anthocyanidin synthase
Pn	Peonidin
Pn3Ga	Peonidin-3-*O*-galactoside
PST	Phenyl sulfotranferases
Pt	Petunidin
RE	Reference electrode
ROS	Reactive oxygen species
RSC	Radical scavenging capacity
SGLT1	Na^+^-glucose transporter
UFGT	UDP-glucose-flavonoid-3-o-glucosyltransferase
UGT	Uridine-5′-diphosphate glucoronosyltransferases
VA	Vainillinic acid
VA-3-Gnd	VA-3-glucoronide
VA-Sft	VA-sulfate
WE	Working electrode
3,4-dOH-BA	Methyl-3,4-dihydroxybenzoate
3RT	Anthocyanidin-3-glycoside rhamnosyl-transferase
4-OH-BA	4-Hydroxy-benzaldehyde
